# Interaction of household air pollution and healthy lifestyle on the risk of sarcopenia: China Health and Retirement Longitudinal Study

**DOI:** 10.1265/ehpm.24-00280

**Published:** 2025-08-21

**Authors:** Tao Zeng, Xinliang Liao, Jie Wu

**Affiliations:** 1 Guangdong Provincial Geriatrics Institute, Affiliated Guangdong Provincial People’s Hospital of Southern Medical University, Guangdong Academy of Medical Sciences, Guangzhou 510080, P. R. China; 2 Guangdong Provincial People’s Hospital Ganzhou Hospital, Ganzhou Municipal Hospital, Ganzhou 341000, P. R. China

**Keywords:** Household air pollution, Healthy lifestyle, Sarcopenia, Interaction, Elderly

## Abstract

**Background:**

Exposure to air pollution and adherence to a healthy lifestyle have been identified to be related to sarcopenia. However, the interactive effects between these two factors remain insufficiently elucidated. The present study was designed to investigate the potential interaction exposure to air pollution with healthy lifestyle on the risk of developing sarcopenia.

**Methods:**

In the retrospective cohort study, all data was extracted from China Health and Retirement Longitudinal Study. Household air pollution was assessed based on the utilization of solid fuels for cooking and heating. A lifestyle score was constructed comprising information on physical activity, smoking, drinking and sleeping time. Multivariate logistic regression model was used to assess the effects of household air pollution and healthy lifestyle score on sarcopenia, separately. We further explored the additive interaction between household air pollution and healthy lifestyle score to sarcopenia using the interaction table developed by T Anderson. Relative excess risk due to interaction (RERI), attributable proportion (AP), and synergy index (SI) were used to evaluate the additive interactive effect.

**Results:**

2,114 participants were included in this study. The result indicated that exposed to household air pollution [adjusted relative risk (RR) = 1.80, 95% confidence interval (CI): 1.15–2.94] and unhealthy lifestyle (adjusted RR = 1.46, 95%CI: 1.04–2.03) were both significantly associated with increased risk of sarcopenia. Furthermore, participants exposed to both household air pollution and an unhealthy lifestyle exhibited a significantly higher risk of sarcopenia relative to those without household air pollution exposure and maintaining a healthy lifestyle (adjusted RR = 2.44). But RERI, AP, and SI suggested that there is no statistically significant additive interaction between household air pollution exposure and healthy lifestyle factors in relation to sarcopenia risk.

**Conclusion:**

Household air pollution in conjunction with an unhealthy lifestyle confers a significantly higher risk of sarcopenia compared to either factor in isolation, with no evidence of a significant additive interaction between these two risk factors.

**Supplementary information:**

The online version contains supplementary material available at https://doi.org/10.1265/ehpm.24-00280.

## Introduction

Sarcopenia is a geriatric syndrome characterized by loss of skeletal muscle mass, low muscle strength, and/or low physical performance [1]. It represents a significant public health challenge confronting aging societies, as it elevates the risks of falls, disability, functional deterioration, frailty, hospitalization, and mortality [2]. According to reports, the prevalence of sarcopenia in Asia’s elderly population ranges from 6.8% to 25.7% [3, 4]. In older adults, sarcopenia typically emerges as an age-related condition that is influenced not only by contemporaneous risk factors but also by genetic and lifestyle factors throughout the life course [5]. Early identification of the factors affecting the occurrence of sarcopenia is of great significance for disease prevention and burden control.

Emissions of air pollutants-including carbon monoxide (CO), sulfur dioxide (SO_2_), and particulate matter (PM)-resulting from the use of solid fuels constitutes significant contributors to indoor pollution and has been linked to a range of adverse health effects [6, 7]. In rural China, traditional solid fuels remain the primary energy source for household cooking and heating [8, 9]. Exposure to air pollution may induce bodily inflammation and oxidative stress, resulting in the reduction of skeletal muscle mass and impairment of its function [10, 11]. Previous research has demonstrated that adopting a healthy lifestyle constitutes a crucial strategy for the prevention and management of chronic diseases [12, 13]. Lifestyle, such as adopting a healthy diet, quitting smoking, and engaging in optimal physical activity are recommended by the World Health Organization (WHO) as cost-effective strategies for preventing non-communicable diseases [14]. Chen et al., found that adherence to a healthy lifestyle might mitigate the risk of cardiometabolic multimorbidity resulting from exposure to household air pollution [15]. Furthermore, adherence to a healthy lifestyle may attenuate the association between depression and sarcopenia risk among individuals aged 45 years and above [16].

Although prior studies have investigated the individual impacts of air pollution and lifestyle factors on health outcomes, limited research has explored their potential interaction effect on sarcopenia. Thus, the present study was designed to investigate the interaction between exposure to household air pollution and healthy lifestyle on the risk of sarcopenia.

## Methods

### Study population

In this retrospective cohort study, all data was extracted from China Health and Retirement Longitudinal Study (CHARLS). The CHARLS database is an ongoing, nationally representative survey of adults aged 45 years and older, conducted by the National School of Development at Peking University. The study encompasses resident populations across 28 provinces in China (https://charls.pku.edu.cn/). The national baseline survey was initially carried out in 2011 and followed up every two years, utilizing a multistage probability sampling design and a probability-proportional-to-size sampling technique [17]. The CHARLS was approved by the Ethical Review Committee at Peking University (approval number IRB00001052-11015), and all participants signed informed consent before participation.

**Fig. 1:**
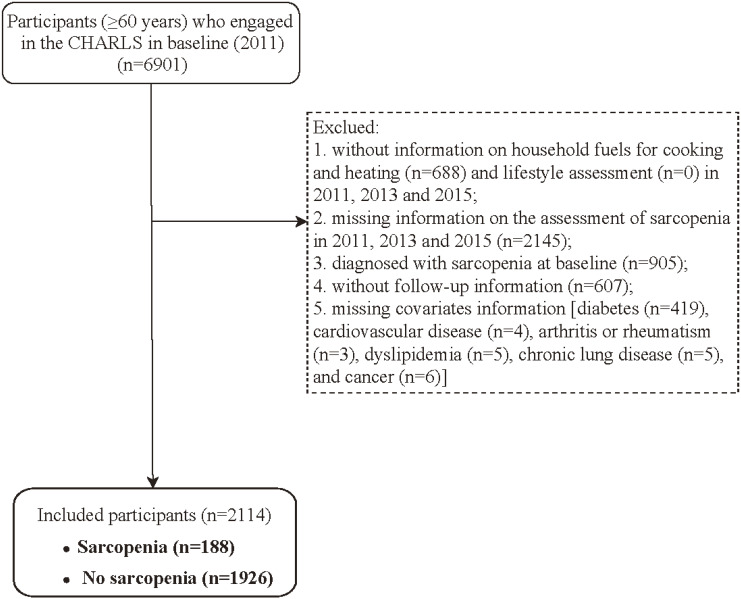
Patient selection flow chart.

In this study, we conducted a retrospective cohort analysis using three waves of CHARLS data. The data employed was from the CHARLS 2011 baseline survey and follow-up in CHARLS 2013 and 2015. All statistical analyses were performed in 2024. Individuals aged ≥60 years old from CHARLS database in 2011 (n = 6901). Exclusion criteria: (1) without information on household fuels for cooking and heating and lifestyle assessment in 2011, 2013 and 2015; (2) missing information on the assessment of sarcopenia in 2011, 2013 and 2015; (3) diagnosed with sarcopenia at baseline; (4) without follow-up information; (5) missing covariates information [diabetes, cardiovascular disease (CVD), arthritis or rheumatism, dyslipidemia, chronic lung disease, and cancer]. Ultimately, a total of 2114 participants were included for further analysis (Fig. 1).

### Data collection

#### Assessment of sarcopenia

Sarcopenia is a progressive and generalized skeletal muscle disorder. In accordance with the Asian Working Group for Sarcopenia (AWGS) 2019 algorithm, sarcopenia was diagnosed when low muscle mass plus low muscle strength or low physical performance are detected [18]. Muscle mass was measured by appendicular skeletal muscle mass (ASM)/height^2^: ASM/height^2^ = [0.193 × weight (kg) + 0.107 × height (cm) − 4.157 × gender (male = 1, female = 2) − 0.037 × age − 2.631]/height^2^. The cut-off value for defining low muscle mass was determined based on the ASM/height^2^ of the study population’s lowest 20th percentile. The assessment of muscle strength involved measuring grip strength, with each participant’s hand being measured twice to determine the average of their highest recorded value. Grip strengths lower than 28 kg for men and 18 kg for women were identified as indicators suggesting low muscle strength. The evaluation of physical performance was determined by assessing gait speed or 5-time chair stand tests. Low physical performance was defined as gait speed on <1.0 m/s, 5-time chair stand test ≥12 s. The primary outcome was the incidence of sarcopenia, which was defined as participants who were classified as having sarcopenia during either of the two follow-up assessments at the four-year follow-up period. Our analysis utilized follow-up data from database 2013 and 2015. Thus, we collected data on grip strength, gait speed, and the 5-time chair stand test from included patients in CHARLS database 2013 and 2015.

#### Assessment of household air pollution and healthy lifestyle

Household air pollution and healthy lifestyle were considered as exposure variables, with data obtained from the baseline survey of the CHARLS 2011 (baseline data). CHARLS participants’ data on household air pollution was assessed based on the use of solid fuels for cooking and heating. The information of solid fuels for cooking and heating was collected using standardized questionnaires by trained interviewers: “What is the main heating energy source?” and “What is the main source of cooking fuel?” Those individuals reporting petroleum gas, natural gas, marsh gas, solar and electricity were defined as using clean fuels. Individuals who reported using coal, crop residue, or wood burning as their primary fuel source of cooking and heating were categorized as using solid fuels, which are considered to have household air pollution [19].

A lifestyle score was constructed by integrating data on physical activity, smoking, drinking and sleeping time [20]. These domains have been used to comprehensively assess the healthy behavior status of middle-aged and older adults [21]. The physical activity score is assigned 0 points if the weekly metabolic equivalent of task (MET·min/week) is <600, and 1 point otherwise. Current smoking status is scored as 0 points if present and 1 point if absent. Similarly, current drinking status is scored as 0 points if present and 1 point if absent. Sleep duration is categorized as 0 points for short (≤6 hours) or long (>9 hours) duration, and 1 point for normal sleep duration. The total lifestyle score is the sum of these individual indicators, ranging from 0 to 4 points, with higher scores indicating a healthier lifestyle. Participants were then stratified based on the median lifestyle score into two groups: an unhealthy lifestyle group (score <2) and a healthy lifestyle group (score ≥2).

#### Assessment of possible covariates

Possible covariates in this study encompassed demographic, examination, laboratory, and questionnaire data: age (years), gender, educational background, marital status, nationality, individual income, residence, self-assessed health status, hypertension, dyslipidemia, diabetes, cancer, chronic lung diseases, cardiovascular disease (CVD), arthritis or rheumatism, anemia, body mass index (BMI, kg/m^2^), C-reactive protein (CRP, mg/L), and estimate glomerular filtration rate (eGFR, mL/min/1.73 m^2^). In this study, anemia was defined according to the World Health Organization (WHO) criteria, which classified individuals with a hemoglobin level below 13 g/dL for men and below 12 g/dL for women as having anemia [22].

### Statistical analysis

Continuous variables with normal distribution were exhibited as mean ± standard error (Mean ± SE), and comparison between groups adopted independent sample t-test or t′-test. Categorical variables were described in terms of number of cases and composition ratio n (%), and the comparison between groups was performed by Chi-square or Fisher’s exact test. We adopted the univariate logistic regression analysis and backward stepwise regression method to explore the possible covariates related to sarcopenia (*P* < 0.05; Supplemental Table 1). Then, the univariate and multivariate logistic regression models were carried out to assess the correlation of exposure to household air pollution and healthy lifestyle score on sarcopenia separately. Model 1 was a crude model without covariate-adjusted (univariate logistic regression model); Model 2 was adjusted for age, nationality, BMI, ASM, grip strengths, self-assessed health status, and dyslipidemia (multivariate logistic regression model). Variance inflation factor (VIF) was calculated to assess the variables collinearity, with VIF less than 10 considered to indicate no significant collinearity (Supplemental Table 2). Relative risk (RR) with 95% confidence interval (CI) was reported. Importantly, we explored the additive interaction between exposure to household air pollution and healthy lifestyle score to sarcopenia using the interaction table developed by T Anderson. The following interaction indices and their corresponding 95% CI were calculated [23]: Relative excess risk due to interaction (RERI), attributable proportion (AP), and synergy index (SI). When the RERI is positive, it signifies an increased risk attributable to additive interaction. The AP represents the proportion of risk attributed to the interaction in the doubly exposed group. The SI can be interpreted as the ratio of the combined increased risk from both exposures to the sum of the individually increased risks [23]. When the 95% CI of RERI or AP contains 0, or the 95% CI of SI contains 1, it indicates that household air pollution and healthy lifestyle do not have a significant additive interaction on sarcopenia risk [24]. For variables with missing data, we categorized them as “Unknown”. All statistical analyses were conducted using R software (version 4.2.3), with a significance threshold set at *P* < 0.05.

## Results

### General characteristics

A total of 2,114 participants were ultimately enrolled in this retrospective cohort study, with a mean (±SE) age of 66.34 (±5.03) years. All participants’ characteristics were shown in Table 1. The study population comprised approximately 52.18% male participants. The overall prevalence of sarcopenia was 8.89% (n = 188). Compared with those with no-sarcopenia, subjects with sarcopenia were more likely to be older and male, live in rural setting, had lower BMI, and had higher prevalence of exposure to household air pollution and unhealthy lifestyle. Detailed baseline information was given in Table 1.

**Table 1:** Characteristics of all participants by sarcopenia status

**Variables**	**Total** **(n = 2114)**	**No sarcopenia** **(n = 1926)**	**Sarcopenia** **(n = 188)**	**Statistics**	***P***
Household environmental pollution, yes, n (%)	1670 (79.00)	1504 (78.09)	166 (88.30)	χ^2^ = 10.152	0.001
Healthy lifestyle score, n (%)				χ^2^ = 8.182	0.004
≥2	1542 (72.94)	1422 (73.83)	120 (63.83)		
<2	572 (27.06)	504 (26.17)	68 (36.17)		
ASM, n (%)				-	<0.001
High	2107 (99.67)	1924 (99.90)	183 (97.34)		
Low	7 (0.33)	2 (0.10)	5 (2.66)		
Grip strengths, n (%)				χ^2^ = 10.103	0.001
High	1746 (82.59)	1607 (83.44)	139 (73.94)		
Low	368 (17.41)	319 (16.56)	49 (26.06)		
Muscle function, n (%)				χ^2^ = 0.751	0.386
High	112 (5.30)	99 (5.14)	13 (6.91)		
Low	2002 (94.70)	1827 (94.86)	175 (93.09)		
Nationality, n (%)				χ^2^ = 12.324	0.002
Han	1506 (71.24)	1370 (71.13)	136 (72.34)		
Other	79 (3.74)	64 (3.32)	15 (7.98)		
Unknown	529 (25.02)	492 (25.55)	37 (19.68)		
Age, years, Mean (±SE)	66.34 (±5.03)	66.13 (±4.96)	68.44 (±5.28)	t′ = −5.753	<0.001
Age, years, n (%)				χ^2^ = 26.741	<0.001
<70	1657 (78.38)	1538 (79.85)	119 (63.30)		
≥70	457 (21.62)	388 (20.15)	69 (36.70)		
Gender, male, n (%)	1103 (52.18)	990 (51.40)	113 (60.11)	χ^2^ = 4.858	0.028
Educational background, n (%)				χ^2^ = 7.192	0.007
Primary school and below	1692 (80.04)	1527 (79.28)	165 (87.77)		
Junior high school and above	422 (19.96)	399 (20.72)	23 (12.23)		
Marital status, Married/Cohabiting, n (%)	1785 (84.44)	1636 (84.94)	149 (79.26)	χ^2^ = 3.795	0.051
Individual income, yuan, n (%)				χ^2^ = 9.567	0.008
<10000	534 (25.26)	483 (25.08)	51 (27.13)		
≥10000	376 (17.79)	358 (18.59)	18 (9.57)		
Unknown	1204 (56.95)	1085 (56.33)	119 (63.30)		
Residence, n (%)				χ^2^ = 6.428	0.040
Rural	1109 (52.46)	997 (51.77)	112 (59.57)		
Urban	249 (11.78)	236 (12.25)	13 (6.91)		
Unknown	756 (35.76)	693 (35.98)	63 (33.51)		
Self-assessed health status, n (%)				χ^2^ = 9.076	0.028
Very Good/Good	339 (16.04)	320 (16.61)	19 (10.11)		
Fair	877 (41.49)	805 (41.80)	72 (38.30)		
Very poor/poor	411 (19.44)	367 (19.06)	44 (23.40)		
Unknown	487 (23.04)	434 (22.53)	53 (28.19)		
Hypertension, yes, n (%)	1091 (51.61)	1010 (52.44)	81 (43.09)	χ^2^ = 5.634	0.018
Dyslipidemia, yes, n (%)	1187 (56.15)	1104 (57.32)	83 (44.15)	χ^2^ = 11.540	0.001
Diabetes, yes, n (%)	370 (17.50)	342 (17.76)	28 (14.89)	χ^2^ = 0.784	0.376
Cancer, yes, n (%)	16 (0.76)	14 (0.73)	2 (1.06)	-	0.647
Chronic lung diseases, yes, n (%)	260 (12.30)	230 (11.94)	30 (15.96)	χ^2^ = 2.202	0.138
CVD, yes, n (%)	372 (17.60)	345 (17.91)	27 (14.36)	χ^2^ = 1.255	0.263
Arthritis or rheumatism, yes, n (%)	789 (37.32)	722 (37.49)	67 (35.64)	χ^2^ = 0.177	0.674
Anemia, n (%)				χ^2^ = 6.415	0.040
No	1517 (71.76)	1393 (72.33)	124 (65.96)		
Yes	199 (9.41)	172 (8.93)	27 (14.36)		
Unknown	398 (18.83)	361 (18.74)	37 (19.68)		
BMI, kg/m^2^, n (%)				χ^2^ = 108.341	<0.001
<24	1229 (58.14)	1052 (54.62)	177 (94.15)		
≥24	885 (41.86)	874 (45.38)	11 (5.85)		
CRP, mg/L, n (%)				χ^2^ = 0.516	0.772
≤3	1375 (65.04)	1257 (65.26)	118 (62.77)		
>3	336 (15.89)	305 (15.84)	31 (16.49)		
Unknown	403 (19.06)	364 (18.90)	39 (20.74)		
eGFR, mL/min/1.73 m^2^, n (%)				-	0.173
<60	44 (2.08)	37 (1.92)	7 (3.72)		
≥60	1666 (78.81)	1524 (79.13)	142 (75.53)		
Unknown	404 (19.11)	365 (18.95)	39 (20.74)		

Abbreviation: ASM, appendicular skeletal muscle; CVD, cardiovascular disease; BMI, body mass index; CRP, C-reactive protein; eGFR, estimate glomerular filtration rate.

### Association between household air pollution or healthy lifestyle score and sarcopenia

As demonstrated in Table 2, the univariate logistic regression analysis revealed that participants exposed to household air pollution exhibited a higher sarcopenia risk (RR = 2.12, 95%CI: 1.37–3.43) compared to those without such exposure. After adjusting for confounding factors, the risk remained elevated, though somewhat attenuated, with household air pollution continuing to serve as an independent risk factor for sarcopenia (adjusted RR = 1.80, 95% CI: 1.15–2.94). In addition, the association between healthy lifestyle score and sarcopenia is shown in Table 2. Model 1 (RR = 1.60, 95%CI: 1.16–2.18; *P* = 0.003) showed that sarcopenia risk in the unhealthy lifestyle group was increased in comparison with the healthy lifestyle group, with similar results in Model 2 (adjusted RR = 1.46, 95%CI: 1.04–2.03; *P* = 0.026).

**Table 2:** Independent effects of household air pollution and healthy lifestyle score on the risk of sarcopenia

**Variables**	**Outcome/Total**	**Model 1**		**Model 2**	

		**RR (95% CI)**	***P***	**RR (95% CI)**	***P***
Exposed to household air pollution					
No	22/444	Ref		Ref	
Yes	166/1670	2.12 (1.37–3.43)	0.001	1.80 (1.15–2.94)	0.014
Healthy lifestyle score					
Healthy lifestyle	120/1542	Ref		Ref	
Unhealthy lifestyle	68/572	1.60 (1.16–2.18)	0.003	1.46 (1.04–2.03)	0.026

Abbreviation: RR, relative risk; CI, confidence interval;

Model 1 was a crude model without covariate-adjusted (univariate logistic regression model);

Model 2 was adjusted for age, nationality, body mass index, appendicular skeletal muscle mass, grip strengths, self-assessed health status, and dyslipidemia (multivariate logistic regression model).

### Interaction between household air pollution and healthy lifestyle score on sarcopenia

**Fig. 2:**
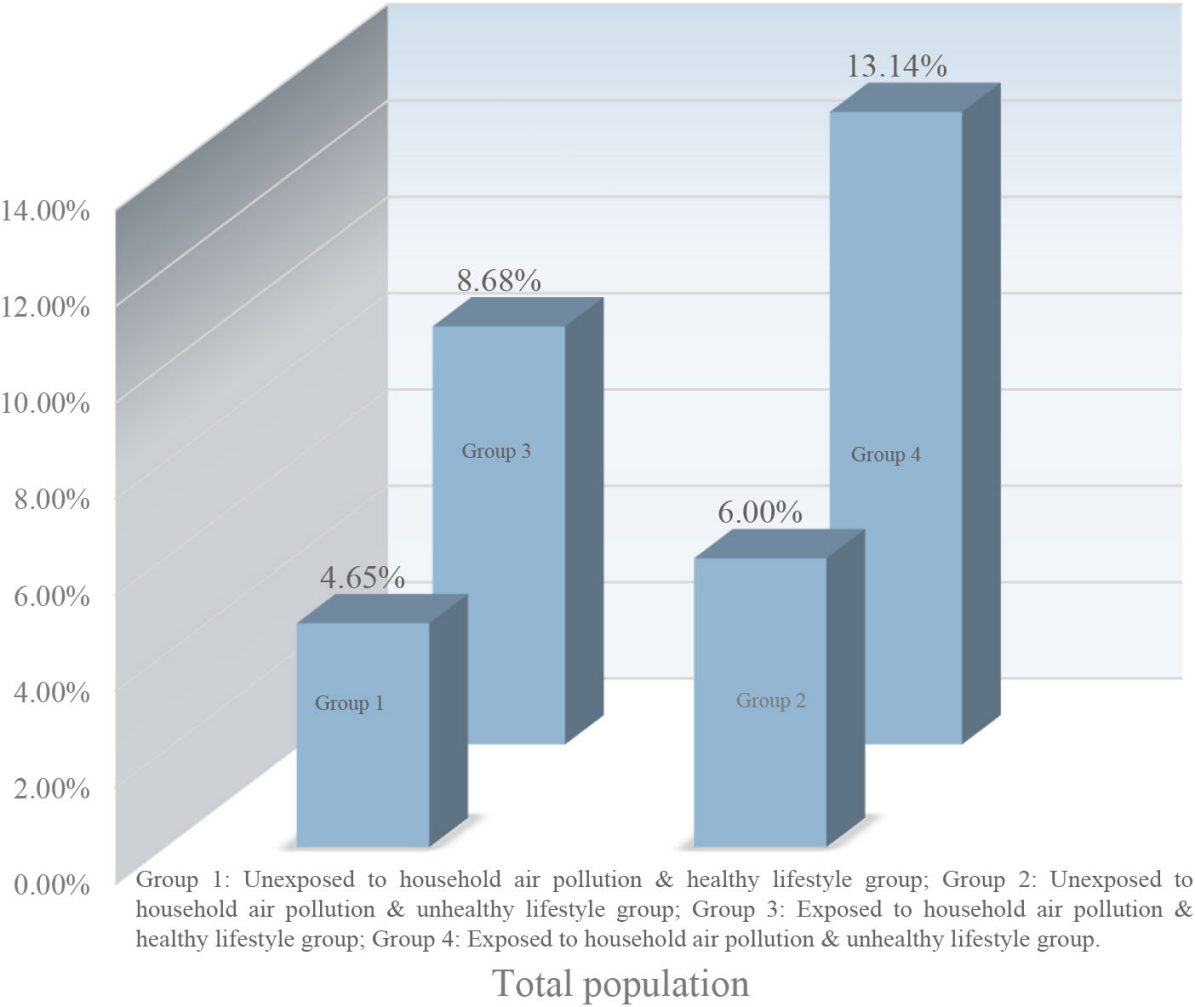
The incidence of sarcopenia in total population.

Participants were divided into four groups: unexposed to household air pollution & healthy lifestyle group; unexposed to household air pollution & unhealthy lifestyle group; exposed to household air pollution & healthy lifestyle group; exposed to household air pollution & unhealthy lifestyle group. Figure 2 shows the prevalence of sarcopenia in the four group of participants. The findings demonstrated that participants exposed to both household air pollution and unhealthy lifestyle factors exhibited the highest prevalence of sarcopenia, with an estimated incidence rate of 13.14%.

**Fig. 3:**
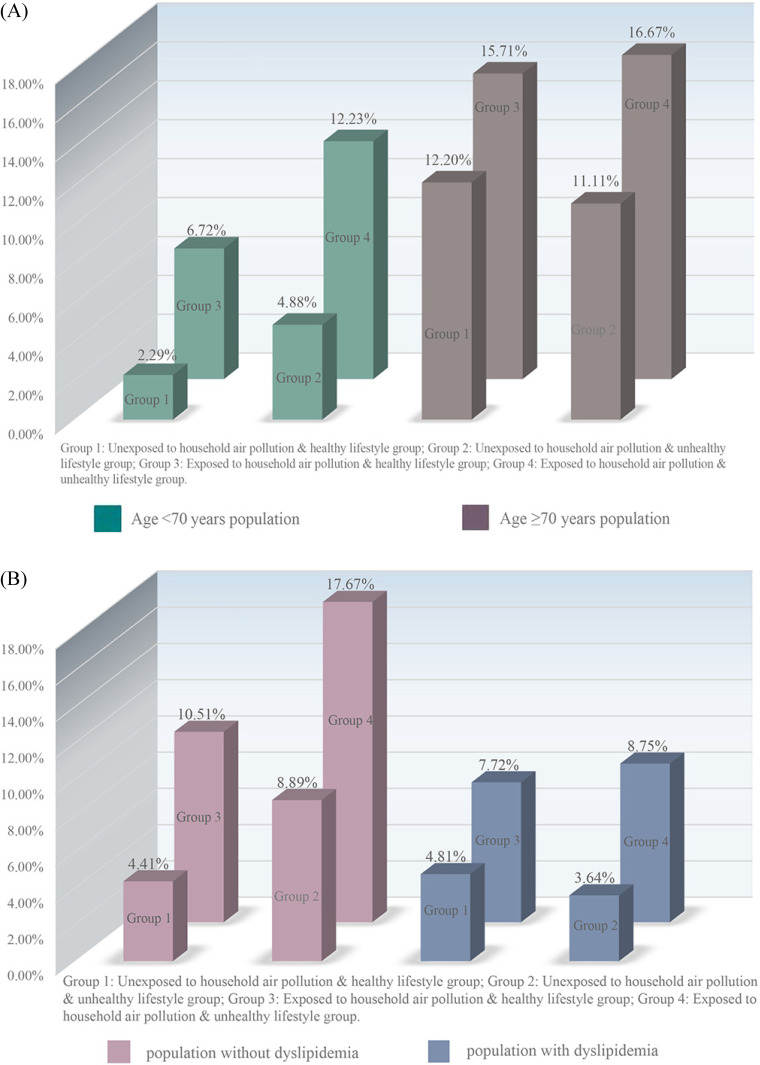
The incidence of sarcopenia in subgroup population: (A) individuals aged <70 years and those aged ≥70 years; (B) individuals with and without dyslipidemia.

In Table 3, participants with both exposed to household air pollution and unhealthy lifestyle had a significantly increased risk of sarcopenia compared to those unexposed to household air pollution and with healthy lifestyle (adjusted RR = 2.44, 95%CI: 1.36–4.39; *P* = 0.003). Although no statistically significant differences were detected, we observed a trend toward increased risk of sarcopenia in both the unexposed to household air pollution with an unhealthy lifestyle group (adjusted RR = 1.25; *P* = 0.658) and the exposed to household air pollution with a healthy lifestyle group (adjusted RR = 1.68; *P* = 0.065), and all *P* for trend = 0.026. Additionally, the results of three indexes (RERI, AP, and SI) indicated no significant additive interaction between household air pollution exposure and healthy lifestyle behaviors on the risk of sarcopenia. As presented in Fig. 3, we found that the participants in the exposed to household air pollution and unhealthy lifestyle group exhibited the highest prevalence of sarcopenia across various subgroup populations.

**Table 3:** Interaction between household air pollution and healthy lifestyle score on the risk of sarcopenia

**Variables**		**Model 1**		**Model 2**	

**Exposed to household air pollution**	**Healthy lifestyle score**	**RR (95% CI)**	***P***	**RR (95% CI)**	***P***
No	Healthy lifestyle	Ref		Ref	
	Unhealthy lifestyle	1.31 (0.50–3.44)	0.585	1.25 (0.47–3.34)	0.658
Yes	Healthy lifestyle	1.95 (1.14–3.35)	0.016	1.68 (0.97–2.92)	0.065
	Unhealthy lifestyle	3.10 (1.76–5.47)	<0.001	2.44 (1.36–4.39)	0.003
*P* for trend		<0.001		0.026	
RERI		0.84 (−0.58–2.26)		0.51 (−0.81–1.83)	
AP		0.27 (−0.18–0.72)		0.21 (−0.33–0.75)	
SI		1.67 (0.53–5.23)		1.55 (0.36–6.75)	

Abbreviation: RR, relative risk; CI, confidence interval; RERI, relative excess risk due to interaction; AP, attributable proportion; SI, synergy index. When the 95% CI of RERI or AP contains 0, or the 95% CI of SI contains 1, it indicates that household air pollution and healthy lifestyle do not have a significant additive interaction on sarcopenia risk.

Model 1 was a crude model without covariate-adjusted;

Model 2 was adjusted for age, nationality, body mass index, appendicular skeletal muscle mass, grip strengths, self-assessed health status, and dyslipidemia.

## Discussion

To the best of our knowledge, this study was the first to utilize nationally representative data in examining not only the independent association between exposure to household air pollution and healthy lifestyle with sarcopenia, but also the joint effect of exposure to household air pollution and healthy lifestyle to sarcopenia in a middle-aged and elderly Chinese population. The findings demonstrated a significant positive association between exposure to household air pollution derived from solid fuel combustion and an elevated risk of sarcopenia, even after adjusting for potential confounding variables. Furthermore, an unhealthy lifestyle was identified as a substantial independent risk factor for increased sarcopenia prevalence. Although the additive interaction analysis did not yield statistically significant results for the interaction term, it is remarkably that participants exposed to both household air pollution and unhealthy lifestyle had a significantly increased risk of sarcopenia compared to those both unexposed to household air pollution and healthy lifestyle. In other words, there may be a joint effect between household air pollution and unhealthy lifestyles to a higher risk of developing sarcopenia, and the joint effect may not be an additive interaction effect.

Mounting evidence showed that the household air pollution caused by solid fuels was harmful to health. Based on our research findings, the effect of household air pollution on sarcopenia development aligns with the results of previous studies. A study involving 31,209 adults aged 50 years and older demonstrated a significant association between exposure to ambient particulate matter 2.5 and household air pollution from domestic solid fuels with decreased hand-grip strength [25]. A cohort study conducted by Jiang et al. further suggested that the utilization of household solid fuel for cooking and heating may increase the risk of sarcopenia incidence [26]. Baseline data from the UK Biobank demonstrated that revealed that exposure to ambient air pollution might be one risk factor of sarcopenia and its components [27].

The precise pathophysiological mechanisms linking air pollution to sarcopenia have yet to be fully elucidated. A possible explanation for this relationship is that air pollutants may trigger oxidative stress, subsequently leading to muscle tissue damage and mitochondrial dysfunction [28, 29]. Long-term exposure to oxidative stress was associated with reduced muscle regeneration and accelerated muscle atrophy [30]. Additionally, air pollution may cause systemic inflammation [31]. The pathophysiological mechanisms underlying sarcopenia are substantially linked to chronic low-grade systemic inflammation, suggesting a plausible association between ambient air pollution exposure and compromised muscle health [32]. Further studies on the mechanism between these factors are needed in the future. Sarcopenia has been reported to be influenced by lifestyle habits, such as reduced physical activity, alcohol consumption, smoking of tobacco, and sleeping time [33, 34]. In this study, healthy lifestyle characterized by physical activity, smoking, drinking and sleeping time was identified as being significantly associated with a reduced risk of sarcopenia. Engaging in appropriate levels of physical activity has been demonstrated to promote mitochondrial biogenesis and improve muscle strength [35]. Smoking exacerbates muscle fatigue and contributes to protein catabolism disorders, resulting in a decline in both muscle mass and function. Adequate sleep essential for facilitating physiological recovery and cellular rejuvenation, while excessive or insufficient sleep can disrupt metabolic functioning, leading to a reduction in muscle mass and physical performance [36].

Additionally, our study implied that exposure to household air pollution combined with an unhealthy lifestyle increased the risk of sarcopenia. It is worth noting that the result should be interpreted with caution owing to the non-statistically significant additive interaction. The underlying mechanism for the joint effect of exposure to household air pollution and unhealthy lifestyle on the higher risk of sarcopenia remains indistinct. The potential explanation lies in the fact that the combination of household air pollution and an unhealthy lifestyle could further aggravate mitochondrial damage [25, 26, 34]. More studies are needed to explore the association of interaction between exposure to household air pollution and healthy lifestyle score to sarcopenia and the exact biological mechanisms. In short, identing the joint effect between household air pollution and unhealthy lifestyle holds significant implications for the prevention and clinical management of sarcopenia. Prevention efforts for sarcopenia should focus on enhancing surveillance of the living environment for elderly individuals, promoting the adoption of clean energy in rural areas, developing targeted strategies for appropriate physical exercise, implementing health education initiatives, or encouraging lifestyle modifications.

However, our study had several limitations. Firstly, the assessment of household air pollution and healthy lifestyle was based on self-reported questionnaire, which might be some memory bias. Additionally, it is impossible to quantify the household air pollution, and this study only used the solid fuels for cooking and heating to reflect the exposure of household air pollution. There may be other possible sources of household air pollution, such as second-hand smoke and formaldehyde from home renovations, which could potentially impact the association between household air pollution and sarcopenia. Secondly, given that the study exclusively involved participants from China, the generalizability of the findings to other countries may be limited. Thirdly, there may still be unincluded confounding factors that could potentially affect the sarcopenia risk, such as nutritional intake and stress management. Nutritional intake could impact the muscle strength gains [37]. Several studies have stated that stress may lead to poor unhealthy lifestyle, such as smoking and drinking [38, 39]. Also, Wang et al., also found that occupational stress was significantly associated with an increased risk of both multiple-site Work-related musculoskeletal disorders and overall Work-related musculoskeletal disorders [40]. Lastly, due to the retrospective cohort study design, we were unable to establish a causal association between exposure to household air pollution, unhealthy lifestyle and sarcopenia. Additional genetic research and clinical trials are necessary to reinforce and validate these findings.

## Conclusion

Overall, exposure to household air pollution from solid fuels, or unhealthy lifestyle was found to be significantly associated with a higher risk of sarcopenia. Although the additive interaction effect did not achieve statistical significance, our findings also indicated that the combined risk of household air pollution and unhealthy lifestyle was significantly greater than their individual effects on sarcopenia risk. Prevention strategies for sarcopenia may be prioritize strengthening environmental surveillance systems for elderly populations, advancing the transition to clean energy sources in rural communities, formulating evidence-based physical activity interventions, executing comprehensive health education programs, and encouraging lifestyle modifications.

## Abbreviations

CO: carbon monoxide
SO_2_: sulfur dioxide
PM: particulate matter
WHO: World Health Organization
CHARLS: China Health and Retirement Longitudinal Study
CVD: cardiovascular disease
ASM: appendicular skeletal muscle mass
CVD: cardiovascular disease
BMI: body mass index
CRP: C-reactive protein
eGFR: estimate glomerular filtration rate
WHO: World Health Organization
RR: Relative risk
CI: confidence interval
RERI: Relative excess risk due to interaction
AP: attributable proportion
SI: synergy index

## Supplementary information

**Additional file 1:** **Supplemental Table 1** The possible covariates related to sarcopenia. **Supplemental Table 2** The assessment of variables collinearity.

## Declarations

### Availability of data and material

The datasets generated and/or analyzed during the current study are available in the CHARLS database, http://charls.pku.edu.cn/.
