# Supplementary material for: Interaction of household air pollution and healthy lifestyle on the risk of sarcopenia: China Health and Retirement Longitudinal Study
Source: Environ Health Prev Med. 2025 Aug 21;30:65. doi: 10.1265/ehpm.24-00280 (PMC12394076; doi:10.1265/ehpm.24-00280)
Supplement: Supplementary file 1 — Additional file 1: Supplemental Table 1 The possible covariates related to sarcopenia. Supplemental Table 2 The assessment of variables collinearity. [file ehpm-30-065-s001.docx]

**Supplemental Table 1 The possible covariates related to sarcopenia**

| **Variables** | **Weighted univariate logistic regression analysis** | | **Backward stepwise regression** | | | |
| --- | --- | --- | --- | --- | --- | --- |
|  |  |  | **Step 1** | | **Step 2** | |
|  | **RR (95% CI)** | ***P*** | **RR (95% CI)** | ***P*** | **RR (95% CI)** | ***P*** |
| ASM |  |  |  |  |  |  |
| High | Ref |  | Ref |  | Ref |  |
| Low | 26.28 (5.62-184.42) | <0.001 | 26.46 (4.82-208.27) | <0.001 | 34.24 (6.27-268.83) | <0.001 |
| Grip strengths |  |  |  |  |  |  |
| High | Ref |  | Ref |  | Ref |  |
| Low | 1.78 (1.25-2.50) | 0.001 | 1.45 (1.00-2.08) | 0.047 | 1.57 (1.09-2.24) | 0.014 |
| Muscle function |  |  |  |  |  |  |
| High | Ref |  |  |  |  |  |
| Low | 0.73 (0.42-1.39) | 0.302 |  |  |  |  |
| Nationality |  |  |  |  |  |  |
| Han | Ref |  | Ref |  | Ref |  |
| Other | 2.36 (1.27-4.15) | 0.004 | 2.48 (1.28-4.54) | 0.005 | 2.34 (1.22-4.24) | 0.007 |
| Unknown | 0.76 (0.51-1.09) | 0.150 | 0.71 (0.47-1.04) | 0.087 | 0.66 (0.44-0.97) | 0.039 |
| Age, years |  |  |  |  |  |  |
| <70 | Ref |  | Ref |  | Ref |  |
| ≥70 | 2.30 (1.67-3.15) | <0.001 | 2.19 (1.55-3.09) | <0.001 | 2.27 (1.63-3.16) | <0.001 |
| Gender |  |  |  |  |  |  |
| Male | Ref |  |  |  |  |  |
| Female | 0.70 (0.52-0.95) | 0.023 |  |  |  |  |
| Educational background |  |  |  |  |  |  |
| Primary school and below | Ref |  |  |  |  |  |
| Junior high school and above | 0.53 (0.33-0.82) | 0.006 |  |  |  |  |
| Marital status |  |  |  |  |  |  |
| Married/Cohabiting | Ref |  |  |  |  |  |
| Other | 1.48 (1.00-2.13) | 0.041 |  |  |  |  |
| Individual income, yuan |  |  |  |  |  |  |
| <10000 | Ref |  |  |  |  |  |
| ≥10000 | 0.48 (0.27-0.81) | 0.009 |  |  |  |  |
| Unknown | 1.04 (0.74-1.48) | 0.829 |  |  |  |  |
| Residence |  |  |  |  |  |  |
| Rural | Ref |  |  |  |  |  |
| Urban | 0.49 (0.26-0.85) | 0.018 |  |  |  |  |
| Unknown | 0.81 (0.58-1.11) | 0.200 |  |  |  |  |
| Self-assessed health status |  |  |  |  |  |  |
| Very Good/Good | Ref |  | Ref |  | Ref |  |
| Fair | 1.51 (0.91-2.61) | 0.124 | 1.60 (0.94-2.84) | 0.095 | 1.63 (0.97-2.90) | 0.078 |
| Very poor/poor | 2.02 (1.17-3.60) | 0.014 | 2.22 (1.25-4.09) | 0.008 | 2.30 (1.31-4.22) | 0.005 |
| Unknown | 2.06 (1.21-3.63) | 0.009 | 2.08 (1.19-3.81) | 0.013 | 2.27 (1.31-4.10) | 0.005 |
| Hypertension |  |  |  |  |  |  |
| No | Ref |  |  |  |  |  |
| Yes | 0.69 (0.51-0.93) | 0.015 |  |  |  |  |
| Dyslipidemia |  |  |  |  |  |  |
| No | Ref |  | Ref |  | Ref |  |
| Yes | 0.59 (0.43-0.79) | 0.001 | 0.66 (0.47-0.94) | 0.020 | 0.60 (0.44-0.82) | 0.002 |
| Diabetes |  |  |  |  |  |  |
| No | Ref |  |  |  |  |  |
| Yes | 0.81 (0.52-1.21) | 0.325 |  |  |  |  |
| Cancer |  |  |  |  |  |  |
| No | Ref |  |  |  |  |  |
| Yes | 1.47 (0.23-5.31) | 0.613 |  |  |  |  |
| Chronic lung diseases |  |  |  |  |  |  |
| No | Ref |  |  |  |  |  |
| Yes | 1.40 (0.91-2.09) | 0.111 |  |  |  |  |
| CVD |  |  |  |  |  |  |
| No | Ref |  |  |  |  |  |
| Yes | 0.77 (0.49-1.15) | 0.223 |  |  |  |  |
| Arthritis or rheumatism |  |  |  |  |  |  |
| No | Ref |  |  |  |  |  |
| Yes | 0.92 (0.67-1.26) | 0.617 |  |  |  |  |
| Anemia |  |  |  |  |  |  |
| No | Ref |  |  |  |  |  |
| Yes | 1.76 (1.11-2.71) | 0.013 |  |  |  |  |
| Unknown | 1.15 (0.77-1.68) | 0.473 |  |  |  |  |
| BMI, kg/m^2^ |  |  |  |  |  |  |
| <24 | Ref |  | Ref |  | Ref |  |
| ≥24 | 0.07 (0.04-0.13) | <0.001 | 0.18 (0.04-0.48) | 0.003 | 0.15 (0.04-0.39) | 0.001 |
| CRP, mg/L |  |  |  |  |  |  |
| ≤3 | Ref |  |  |  |  |  |
| >3 | 1.08 (0.70-1.62) | 0.707 |  |  |  |  |
| Unknown | 1.14 (0.77-1.65) | 0.496 |  |  |  |  |
| eGFR, mL/min/1.73m^2^ |  |  |  |  |  |  |
| <60 | Ref |  |  |  |  |  |
| ≥60 | 0.49 (0.23-1.22) | 0.093 |  |  |  |  |
| Unknown | 0.56 (0.25-1.46) | 0.199 |  |  |  |  |

Abbreviation: ASM, appendicular skeletal muscle; CVD, cardiovascular disease; BMI, body mass index; CRP, C-reactive protein; eGFR, estimate glomerular filtration rate; RR, relative risk; CI, confidence interval.

**Supplemental Table 2 The assessment of variables collinearity**

| **Variables** | **VIF** |
| --- | --- |
| ASM | 1.00399 |
| Grip strengths | 1.01777 |
| Nationality | 1.00434 |
| Age | 1.02141 |
| Self-assessed health status | 1.00160 |
| Dyslipidemia | 1.00861 |
| BMI | 1.01018 |

Abbreviation: ASM, appendicular skeletal muscle; BMI, body mass index; VIF, variance inflation factor.
